# New solutions for antibiotic discovery: Prioritizing microbial biosynthetic space using ecology and machine learning

**DOI:** 10.1371/journal.pbio.3003058

**Published:** 2025-02-28

**Authors:** Marnix H. Medema, Gilles P. van Wezel

**Affiliations:** 1 Bioinformatics Group, Wageningen University, Wageningen, The Netherlands; 2 Institute of Biology, Leiden University, Leiden, The Netherlands

## Abstract

A major challenge in natural-product-based drug discovery is the identification of gene clusters most likely to specify new chemistry and bioactivities. This Perspective discusses the challenges and state of the art of antibiotic discovery.

## Teaser

With the explosive increase in genome sequence data, perhaps the major challenge in natural-product-based drug discovery is the identification of gene clusters most likely to specify new chemistry and bioactivities. We discuss the challenges and state-of-the-art of antibiotic discovery based on ecological principles, genome mining and artificial intelligence.

Antibiotics have made a huge contribution to the extension of human life span. However, antimicrobial resistance (AMR) is making existing antibiotics ineffective. At the same time, lack of new antibiotic classes and limited commercial incentives frustrate antibiotic research and development. The converging problems of AMR and a nearly empty discovery pipeline form an impending crisis, often referred to as the “silent pandemic”, which may cause 10 million deaths annually by 2050 [1]. Microorganisms produce a wealth of bioactive secondary metabolites, many of which are used in the clinic. However, no new antibiotics have entered the market since daptomycin was introduced in 2003. One major bottleneck is that of replication, in other words, the fact that antibiotic discovery pipelines lead to rediscovery of the same compounds. Then the question is, can we still find new chemical entities that will serve as the drugs of the future? The answer is as simple as it is complicated: if we are to discover new classes of antibiotics, we need to do things that have not been done before.

Whole genome sequencing has revealed that genomes of Actinobacteria contain dozens of biosynthetic gene clusters (BGCs) that encode the synthesis of natural products [2]. Bioinformatic tools such as antiSMASH allow the automated identification of these BGCs and prediction of the classes of molecules they produce. Millions of BGCs have been sequenced that encode the biosynthesis of many thousands of natural product scaffolds, and a recent study estimated that only some 3% of the natural product structural classes have thus far been experimentally characterized [3]. A major question is how to prioritize from the millions of unknown BGCs and their cognate metabolites. Finding ways to activate cryptic or silent BGCs and the application of artificial intelligence are two promising avenues to address this question.

The control of BGCs is tied to the ecological conditions in which antibiotic production evolved [4]. Understanding the underlying mechanisms may open up completely new biology and chemical diversity. The chemical composition of the bacterial habitat and the interactions with other bacteria, plants or insects are likely major determinants. Hosts harness symbiotic microbial products for protection against diseases; when plants or animals are challenged by infections or pests, their stress signals are perceived by microbes in their microbiome, which respond by producing protective bioactive molecules [5]. Elucidating the triggers and cues that activate BGC expression will allow the development of rational elicitation approaches [4], while high-throughput elicitor screens form an attractive alternative [6].

To rationally design elicitors and predict what is needed to activate BGCs of interest, we need to vastly expand the knowledge of the transcription factor regulatory networks (TFRNs) in (Actino)bacteria and how they control BGCs. Only about 5% of all TFs have been characterized in model antibiotic producers such as *Streptomyces*. DNA affinity purification sequencing (DAP-seq) allows generating genome-wide DNA binding profiles of many TFs in high throughput [7] and we expect this technology to hugely expand our knowledge of the TFRNs in Actinobacteria in the coming years. This will accelerate the predictive power of how BGCs are controlled, enabling prediction of how they are activated and thus the characterization of their cognate metabolites. In addition, TFRNs may be harnessed to predict the function of BGCs; for example, the presence of binding sites for the iron master regulator DmdR1 implies that the natural product produced by the BGC is likely to be involved in iron homeostasis [8]. Another way to avoid the regulatory constraints on BGCs is by cloning: placing them under the control of strong and/or artificial promoters, and expressing them in a suitable heterologous host. However, until this procedure is amenable to high(er) throughput, it will only remain applicable to the expression of specific BGCs of interest.

Another approach that has considerable potential to revive natural product discovery is the use of machine learning and artificial intelligence [9]. First, such algorithms can be utilized for improvement of known antimicrobials to enhance their pharmacology or evade resistance mechanisms. This opportunity was recently discussed in detail in another opinion paper in this journal [10]. Besides the generation of large libraries of relatively simple antimicrobial peptides, as discussed there, recent developments in synthetic chemistry, cell-free biosynthesis and engineering make it feasible to rapidly explore natural-product chemical space around many other scaffolds of interest. Important examples are the successful biosynthetic engineering of non-ribosomal peptide synthetases and polyketide synthases [11,12]. Many abandoned antimicrobials could be revisited in this way, or existing antibiotics such as daptomycin and polymyxins could be diversified to evade resistance and reduce toxicity.

Such algorithms also allow the discovery of new scaffolds by finding novel classes of BGCs. Recently, we developed decRiPPter [13], an artificial intelligence approach specifically designed to identify novel classes of ribosomally synthesized and post-translationally modified peptides (RiPPs). This algorithm recognizes potential genes for RiPP precursors primarily based on genomic context, generic sequence characteristics and physicochemical properties. Over 40 putative novel RiPP biosynthetic classes were identified in the genus *Streptomyces*, one of which was validated experimentally as representative of a novel class of lanthipeptides (class V) [13]. DecRiPPter may be the first AI-based algorithm to directly identify a novel subfamily of natural products that includes antibiotic compounds, and we anticipate that many will follow. For example, AI-based mining of the human microbiome for non-modified antimicrobial peptides led to the discovery of promising hits [9]. With AI still in its infancy, we expect that huge discoveries lie ahead of us.

Novel scaffolds can, of course, also be identified using activity-first approaches. Arguably, the most effective way to distinguish which biological activities are caused by novel molecules would be to reliably link them to their cognate BGCs and tandem mass spectra. However, to do so at sufficient scale has thus far been impossible because paired genome, metabolome and bioactivity data of large strain collections is scattered across diverse resources, sometimes inaccessible due to intellectual property (IP) restrictions. A new machine learning technology called ‘federated learning’ has recently emerged that facilitates the identification of patterns across datasets without the need to share the datasets, thus safeguarding IP while enabling large-scale collaborative drug discovery [14]. We believe that by connecting public data with strains available in (semi-)private strain collections, an economically feasible model could be built for future collaborative discovery and development of new antimicrobial drugs.

In general, more intensive collaborative efforts across both academia and industry are needed. Rich integrated datasets resulting from this could fuel global, publicly accessible antibiotic discovery engines and collaborative networks within or between continents, such as the African drug discovery network H3D and the recent Scientific Community for the Discovery of Future Medicines (C4D). The future of antibiotic discovery and development depends as much on new and closer ways of collaboration as on novel technologies. Once these go truly hand in hand, scientists will be able to harness the full biosynthetic space of microbes, which provides real hope of turning the tidal wave of AMR.
